# Searching for “transformative moments”: A qualitative study of nurses’ work during home visits to COPD patients and their caregivers in Norway

**DOI:** 10.1002/nop2.209

**Published:** 2018-10-09

**Authors:** Gunvor Aasbø, Ellen Kristvik, Kari Nyheim Solbrække, Anne Werner

**Affiliations:** ^1^ Health Services Research Unit Akershus University Hospital Lørenskog Norway; ^2^ Institute of Health and Society University of Oslo Oslo Norway

**Keywords:** ambulatory nursing, chronic illness, family caregivers, Mattingly, pulmonary rehabilitation, qualitative research, self‐management support, transformative moments

## Abstract

**Aim:**

The integration of families into healthcare services is being emphasized increasingly in healthcare polices. The aim of this study was to investigate how professionals during home visits support both patients and caregivers in accepting and accommodating to illness.

**Design:**

An explorative qualitative design.

**Methods:**

Participant observations from home visits (*N* = 20) of pulmonary ambulatory nurses to COPD patients in Norway, followed by interviews with these nurses.

**Results:**

Our findings demonstrate the delicate nature of nurses’ work during home visits to COPD patients and their caregivers. They support both patients and caregivers in reconciling themselves with the negative consequences of illness, as well as giving legitimation to and potential room for, sustainable arrangements within the scope of the relationship. The nurses address significant issues, having transformative potential concerning attitudes and practices related to the management of illness and adjusting to a complex illness trajectory.

## 1 INTRODUCTION

In recent decades, there has been increasing emphasis in healthcare policies and practice worldwide on avoiding hospital admissions (Shepperd et al., 2016). Thus, the patient's home has become an increasingly important setting for the provision of healthcare services. Various hospital‐at‐home models and ambulatory services are regarded as important in the provision of coordinated, tailored care for a growing population of chronically ill persons (Bourbeau & Saad, 2013; Ministry of Health & Care Services, 2013). These integrated care services often include aspects of rehabilitation, self‐management support and patient education.

### 1.1 Background

Chronic obstructive pulmonary disease (COPD) is a progressive and incurable illness caused mainly by tobacco smoking (Mannino & Buist, 2007). It is characterized by the obstruction of lung airflow, which interferes with normal breathing. COPD is projected to become the third leading cause of death and the fifth leading cause of morbidity worldwide by 2020. The trajectory of COPD tends to include acute and life‐threatening exacerbations followed by stable phases with some level of recovery.

Pulmonary rehabilitation and self‐management support are recommended for patients with COPD (McCarthy et al., 2015; Spielmanns et al., 2016; Zwerink et al., 2014). The aim of pulmonary rehabilitation is to enable patients to adjust to changes in their levels of function due to illness or disability. Self‐management support involves teaching patients problem‐solving skills with the aim to make them more self‐reliant (Bourbeau & Saad, 2013; Efraimsson, Hillervik, & Ehrenberg, 2008). Rehabilitation and self‐management support have positive effects on patients’ health‐related quality of life (Lenferink et al., 2017; McCarthy et al., 2015; Puhan, Gimeno‐Santos, Cates, & Troosters, 2016; Zwerink et al., 2014). They relieve dyspnoea and fatigue (McCarthy et al., 2015) and are associated with lower probability of hospital admissions (Lenferink et al., 2017; Zwerink et al., 2014). Some researchers have emphasized that rehabilitation programmes and professional self‐management support for patients must include family‐centred, relational care focused on caregivers and the patient–caregiver relationship (Jónsdóttir, 2013; Lake & Staiger, 2010).

A growing body of literature sheds light on the burden of caregivers of COPD patients, which can include negative impacts on physical and mental health, such as fatigue, exhaustion (Simpson, Young, Donahue, & Rocker, 2010; Spence, Hasson, Waldron, & Kernohan, 2008), anxiety and depression (Gabriel, Figueiredo, Jácome, Cruz, & Marques, 2014; Grant, Cavanagh, & Yorke, 2012; Jácome, Figueiredo, Gabriel, Cruz, & Marques, 2014; Lindqvist, Albin, Heikkilä, & Hjelm, 2013). Other challenges caregivers face include the unpredictability of the illness trajectory, patients’ negative attitudes and behaviours and a lack of information from healthcare professionals (Hasson et al., 2009; Spence et al., 2008). Professional information and support are important for caregivers to overcome these barriers and perform the caregiver role while maintaining their own health (Bergs, 2002; Caress, Luker, Chalmers, & Salmon, 2009; Hasson et al., 2009; Lindqvist et al., 2013). However, the view of patients’ and caregivers’ needs as essentially individual and separate has faced criticism (Chattoo & Ahmad, 2008; Seddon & Robinson, 2015).

Ambulatory nursing practice differs considerably from the structured work environment of the hospital. To date, ambulatory services have been evaluated primarily in terms of the economic and clinical outcomes for patients and services (Shepperd et al., 2016; Wong et al., 2008). With a few exceptions, for example, Sahlsten, Larsson, Sjöström, and Plos (2009), Ingadóttir and Jónsdóttir (2010), Leine, Wahl, Borge, Hustavenes, and Bondevik (2017), little effort has been made to understand the role of professionals’ engagement in dialogues with patients and their families to provide both individual and relational support. To our knowledge, the ways ambulatory nurses’ work during home visits to support *both* patients and caregivers remains unexplored.

### 1.2 Theoretical perspective

In this study, we have drawn on the work of Cheryl Mattingly (1994, 1998; Mattingly & Lawlor, 2001). Her perspective was developed through observational studies of occupational therapists’ work. Mattingly describes how occupational therapists actively develop “transformative moments” that will make patients desire to act. According to Mattingly, desire structures action and engaging patients in therapy may encourage them to act which in turn may bring about a transformation in the ways of managing and living with illness and disability (Mattingly, 1994; Tropea, 2012). In a later study, Mattingly and Lawlor (2001) explore the significance of healthcare professionals’ conveyance recognition through common activities, interaction and support. They suggest that the interaction between professionals, patients and caregivers may reveal possible worlds and possible selves. In relation to our study, Mattingly's perspective offers a fruitful lens through which to explore how nurses interact with patients and their caregivers.

### 1.3 Aim

The aim of this study was to investigate how ambulatory nurses work during home visits to COPD patients. Specifically, we attempted to improve understanding of the work nurses do to support both patients and their caregivers in illness management, as well as to initiate processes of motivation and change to accommodate illness.

## 2 THE STUDY

### 2.1 Design

This qualitative study has an explorative, inductive and deductive research design: it is both empirically driven and theoretically informed, following the principles of thematic analysis outlined by Coffey and Atkinson (1996).

### 2.2 Setting

The empirical context of our study is two nurse‐led ambulatory services organized at the pulmonary outpatient clinics of two different hospitals near Oslo, Norway, with 240 and 160 patients in their patient pools, respectively. The patients were diagnosed with severe or very severe COPD and most of them used oxygen treatment at home.

Pulmonary nurses from the ambulatory services provided home visits to patients for education and surveillance purposes once or twice per year. The nurses had the flexibility to visit more or less often to meet individual needs for counselling and monitoring of treatment, as based on the progression of the illness. In the case of both services, the home visits involved monitoring oxygen treatment, addressing issues related to coping in everyday life and managing exacerbations.

### 2.3 Material and data generation

We conducted a qualitative study based on observations and interviews. The first author accompanied three ambulatory nurses to 20 home visits to COPD patients (14 women and 6 men), each of which lasted between 30 min and 120 min (mean = 80 min). At 15 of the home visits, the patients’ spouses were present. The patients were 45–90 years with varying socioeconomic backgrounds. Two of the patients were immigrants.

In line with Mays and Popes’ (1995) recommendation for observational studies, significant situations and conversations were recorded by taking brief notes during the sessions. In the observations, the researcher focused on the interaction between the nurse, patient and caregiver in the home visit, examining how they addressed, discussed and negotiated issues related to illness management. Detailed accounts of each visit were written as soon afterwards as possible.

The first author also interviewed the four nurses who operated the two ambulatory services. They were women in their fifties or sixties and had lengthy experience of working in pulmonary departments. Three of the nurses were specialist pulmonary nurses. The interviews took place individually and lasted between 75 and 100 min. They were transcribed verbatim by the first author. A semi‐structured approach was taken, with emphasis being placed on the content of the nurses’ work, the issues they addressed during home visits and the possibilities that arose as a result of meeting patients and caregivers at home.

### 2.4 Analysis

The data analysis was based on the principles of thematic analysis outlined by Coffey and Atkinson (1996). Codes and concepts were generated through a mixed data‐reduction and data‐complication approach. The analysis of the observations and interviews revealed that the nurses faced several challenges related to how to support patients’ and their caregivers’ management of illness. Nevertheless, the caregiver's presence often helped the nurses create opportunities to support the patients. By comparing the observations, we identified two significant patterns of interactions in how the nurses facilitated the processes of change and motivation in the patients and caregivers: (a) adjustment to an altered life situation; and (b) reconciliation to a deteriorating illness. Mattingly's perspective made us attentive to how the nurses actively engaged the patients and caregivers, searching for significant experiences with transformative potential. For this article, we selected for detailed analysis two home visits we considered well suited to illustrate the patterns of interactions when the caregivers also attended.

### 2.5 Rigour

To enhance the strength and reach of the knowledge generated, the concepts of trustworthiness and transferability, described by Lincoln and Cuba (1985), were used to guide the study. The research team regularly met and discussed general patterns running throughout the data, as well as how these particularities and details in the two chosen cases of home visits shed light on the complex interactions among the nurses, patients and caregivers. The transferability of the results was enhanced using detailed, illustrative excerpts from the data and by generating understandings through applying theoretical concepts to the interactions, as Payne and Williams (2005) suggested.

To enhance the trustworthiness and transferability of qualitative research, reflexivity, or awareness to how the researchers and the research process shape the generation of knowledge, was crucial. Observing and rendering the observations reflected situated interpretations; they were not unmediated (Alvesson & Sköldberg, 2018). In particular, the observations carried out challenged our preconception and concern that infrequent visits made it difficult for the nurses to initiate trustful discussions. However, during the observations, we realized the opposite: the conversations with the nurses offered opportunities for the patients and caregivers to discuss significant concerns. In prolongations, the perspective of Mattingly (1994, 1998, Mattingly & Lawlor, 2001) guided us to observe the nurses’ taken‐for‐granted practices and interactions, which made us attentive to how the nurses used the possibilities that arose in the conversations to open up their understandings of illness management.

### 2.6 Ethics

The Regional Committee for Medical and Health Research Ethics in Norway concluded that the study was not under the remit of the Health Research Act (ref. 20101966a). Approval was given by the privacy ombudsman at Akershus University Hospital HF (ref. 2010/042). The nurses informed the patients and caregivers about the study, asking them to participate. Written informed consent was obtained from the participants before data collection. All the names used are pseudonyms.

## 3 RESULTS

In the interviews, the nurses described that meeting the patients at home made it easier to understand their actual situations, the challenges they faced and their support needs. The nurses expressed uncertainty and ambivalence about how to integrate the caregivers into these interactions. According to the nurses, the caregivers and patients may have different and conflicting understandings of the challenges related to illness management. The caregivers thus could be both a resource and a challenge to the nurses’ work. They could serve as a brake in the conversation among the three as the patients often were not “that honest and don't dare to admit things,” as one nurse put it, although emphasizing this was not characteristic for all the relationships.

The observations gave insights into *how* the nurses worked to support the patients and caregivers in illness management. The topics discussed in each visit varied depending on the current individual challenges faced by the patients and caregivers. Issues such as medication adherence, external support, respite, nutrition, physical activity and action plans for managing acute exacerbations at home were often discussed. We chose to describe two home visits in detail to describe the different ways the nurses related to the challenging relationship between the patients and their spouses/partners. The interactions gave insights into the challenges the nurses faced but also the possibilities the discussions with the patients and caregivers gave the nurses in discussions of illness management.

As the interactions described in the following illustrate, engaging both patients and caregivers was crucial to recognize the challenges of both and to generate motivation and legitimacy for adjustments and acceptance.

### 3.1 Adjustment to an altered life situation

Diana was a hairdresser in her 60s. She was a lively woman who loved her work and she still had customers. She was currently recovering from two acute, life‐threatening exacerbations of her disease. The nurse had visited Diana a couple of weeks before without her husband's presence. At that time, Diana had expressed difficulty accepting and managing her illness but displayed openness to counselling. The prior discussion had centred on what changes she had to make to manage her illness; however, counselling Diana at this visit appeared to be more complex with the presence of her husband. On several occasions, the interactions between Diana and her husband indicated that her illness represented tensions between them:

The nurse asked Diana what she believed to be the reason for getting hospitalised. Diana said that on the first occasion, she had pushed herself too hard to keep an appointment with a customer. She could not sleep the night before. Her husband added that she had taken so many sleeping pills and other sedatives that she became listless the day after. He felt that her GP prescribed too much medication. Diana replied: “You only believe in your own points of view!” Her husband answered: “I must be allowed to have opinions”. Diana said that when she realised that she had to cancel the appointment, she collapsed. The nurse said: “If you wish to live longer, what changes are you prepared to make? You can't go on like this. How can you care for yourself?” Diana replied, “I'm happy when I can help others”. They discussed how she could prioritise her energy. Her husband emphasised several times that she would benefit from more physical exercise. The nurse confirmed that she would get stronger if she exercised, but it was also important for Diana to rest. Diana listed activities that were important to her: hairdressing, family and grandchildren, shopping, art museums, physical exercise, gardening. Her husband said that Diana would not manage to quit hair dressing. He added: “She's a magician as [a] hair dresser”. Diana said that she grieved about giving up things that were important to her. Prioritised, Diana wished to exercise, do hair cutting and spend time with her family. Then the nurse made a joke, asking: “And the husband as number five!?”

This example displays how the nurse supported Diana's ongoing work of accepting her illness and adjusting her activities to be able to manage it in various ways. The nurse made efforts to get Diana back on the positive track from the previous visit. This was challenging as Diana's husband's version of why she fell acutely ill provoked Diana. This dynamic was in line with what was observed at most visits: discrepancies were evident in the caregivers’ opinions on how the patients should manage their illnesses and what the patients actually did.

The nurse helped Diana to develop acceptance of her illness and the desire to make important changes to manage it. To give priority to activities that could maintain her sense of self and to give up activities less important in that respect was crucial. Moreover, adjusting the activities in such a way that she could maintain them was also important. But, Diana's husband's input complicated supporting Diana in this process. He had another view about how she should prioritize things and he attempted to make the nurse give legitimacy to his opinion that Diana should prioritize physical exercise. The nurse partly supported his view. The nurse opened a dialogue about Diana's options, emphasizing the importance of finding a better balance between activity and rest. Yet, Diana's husband's contribution made possible that the challenges Diana faced were addressed and understood by the nurse. At other home visits, the nurses addressed various possible or necessary changes to ease and facilitate current and future illness management for the patients and caregivers. However, many issues the nurses discussed with the patients and caregivers were unresolved and, to some extent, created conflicts between them (e.g. travelling, modifying lifestyle, moving to an adjusted flat and participating in rehabilitation).

In the home visit to Diana and her husband, the discussion culminated with the nurse making a joke. The joke seemed to communicate goodwill towards the husband by pointing out that Diana was at risk of forgetting him. Moreover, the joke reflected the balance work that the nurse did of attending to both the patient and the caregiver. The nurse showed recognition of the tensions between them and still she managed to maintain cooperation with both. This particularly shows that the nurse understood how sensitive and difficult it was to adjust to and manage an altered life situation for both the patient and caregiver.

### 3.2 Reconciliation to a deteriorating illness

Tom, who was in his 70s, had severe COPD and had been ill for several years. It had been 1.5 years since the nurse visited him. Tom rarely left his flat anymore, and his asthmatic breath was prominent and made his illness importunate. Tom's wife increasingly participated throughout the conversation:

The nurse asked how he had been since the last visit. Tom said he was very exhausted. He used oxygen, which according to instructions, was supposed to be level three in rest and four in activity. “But what's activity?” he asked. The nurse explained that exhaustion of all kinds is activity. “Getting up in the morning is very exhausting”, he replied. The nurse confirmed that as activity, along with eating and going to the toilet. “Going to the loo?” the wife asked surprised. “You have no idea how many admissions we get from the loo”, the nurse answered. The wife said that he sometimes was very exhausted after having been to the toilet. After an intense cough attack, the nurse asked if he used flutter or a PEP (Positive Expiratory Pressure) device. Tom confirmed that he did, but his wife protested. The nurse said that some patients use it for an hour every morning to expel mucus. This was perhaps exhausting in the beginning, but efficient in the long term. It could improve his breathing and prevent infections. The wife assured the nurse that they would make a greater effort from now on. Addressed to the researcher, she said: “I've encouraged him many times, but I can't nag either”. Later on in the conversation, the nurse asked them if they had any arrangements for relief. Tom answered that he stayed at the local nursing home for two weeks every second month. He added that he would prefer to stay at home, but this way his wife could get some respite. The nurse praised this arrangement: “A lot of patients don't realise the need for this”.

Clarifying what Tom may regard as activity, the nurse found an opportunity to point out “new” issues to be aware of and consider: to acknowledge that going to the toilet had become not only exhausting, but also a potential risk of breathlessness that involved effort, planning and timing. The responses of the patient and the caregiver suggest the importance of giving counselling about the mismatch between the progression of illness and their understanding of and accommodation to it. In other home visits, the nurses explained that emotional reactions could be exhausting for COPD patients; therefore, they are often actively avoided. Such information offered patients and caregivers a new understanding and acceptance of the illness and its implications.

Tom's intense cough attack during the visit gave the nurse an opportunity to address his management of the illness. His wife's correction about his use of flutter exposed him as someone unwisely managing his illness; it gave the nurse scarce but important information that opened up an opportunity to encourage him to invest energy and time in expelling mucus. The nurse's explanation gave the wife legitimacy to further push her husband despite his resistance. This interaction is one of the common ways nurses contribute to enhancing patients’ and caregivers’ shared understanding of illness management. Furthermore, in other observed visits, caregivers rarely addressed directly their experiences of challenges and strain due to their partners’ illness. Issues such as being affected by their partners’ breathlessness were seldom brought up in the home visits by the caregivers themselves. Thus, this indicated that to provide recognition of the severe implications of illness on both patients and caregivers was crucial.

At the end of the visit to Tom and his wife, the nurse used an opportunity to support crucial respite for Tom's wife. Tom acknowledged the necessity for this, but he also communicated displeasure about this arrangement. Considering such an arrangement involves weighing her needs for respite over his feelings of discomfort, Tom's statement easily may have made his wife feel guilty. The nurse acknowledged that his discomfort at the nursing home was a price to pay, but rather than pitying him she emphasized the generosity act on his part. In this way, she seemed to give Tom recognition for caring and contributing to the maintenance of their relationship.

## 4 DISCUSSION

Our findings demonstrate the delicate nature of nurses’ work during home visits to COPD patients and their caregivers. Through recognizing both patients’ and caregivers’ experiences, the nurses manage to initiate discussions of challenging issues. The nurses actively search for significant experiences with transformative potential, through which they help patients and caregivers to not only accept, but also adjust to an altered life situation and complex illness trajectory.

### 4.1 Searching for transformative moments

The visits illustrate the ways nurses use what we, referring to Mattingly's perspective (1994, 1998; Mattingly & Lawlor, 2001), term “transformative moments” to contribute to the creation of a shared understanding between patients and caregivers of illness management. Mattingly and Lawlor (2001) emphasize the importance of creating *activities* that are imaginatively rich enough to convey the moral that, despite a lost or broken body, a self‐worth striving for still exists. During one nurse's conversation with Diana and her husband, the prioritization and adjustment of activities was negotiated so that the patient could find ways to both maintain her sense of self and manage her illness in a sustainable way. Agreeing on activities that were meaningful enough to Diana was important. Diana's husband enabled the nurse to understand the support that his wife needed and to find methods through which the management of her illness could be integrated into her on‐going life project. The nurse enacted understanding of both partners’ needs and feelings through recognition, advice and humour, thereby also supporting them as a couple. Transformative moments led to a shared understanding of the possibilities for maintaining a challenged self.

During the conversation with Tom and his wife, an individual management of his symptoms was expanded to address illness management in the context of their relationship. The nurse actively searched for transformative moments by enhancing the patient's and the caregiver's awareness of the situation, while also respecting the limited capacity that Tom showed for improving his symptom management. Tom's acceptance of being at the nursing home was reconstructed through the nurse's recognition of his action as a caring act. Moreover, by communicating acceptance of his wife's needs, the nurse reinforced Tom's sense of a moral self. This illustrates the importance of nurses addressing and recognizing the needs of both patient and partner. The process of identifying, clarifying and legitimating personal needs is challenging to a relationship and often leads to tensions between husband and wife. These forms of legitimization and reconstruction of meaning can contribute to a shared understanding of the illness and how it affects both.

### 4.2 The home as a healthcare setting: possibilities for relational support

Our findings indicate that in the context of ambulatory nursing, a relational approach also forms a good basis for working collaboratively with both patients and caregivers, a need also recognized by other researchers (Jónsdóttir, 2013; Røthing, Malterud, & Frich, 2015; Sims‐Gould & Martin‐Matthews, 2010). During home visits, nurses’ work is much more flexible and open than their work in hospitals, where services are highly structured with predefined tasks and routine procedures and monitoring and follow‐up take place in a given time frame (Allen, 2015). Home visits offer the opportunity to talk to one patient at a time without distraction, which hospital routines rarely allow (Leine et al., 2017; Wang, Haugen, Steihaug, & Werner, 2012).

As Mattingly (1994, 1998; Mattingly & Lawlor, 2001) developed her perspectives in different rehabilitation contexts, she came to see such transformations as continuing processes unfolding over time. In the context of our study, the clinical encounters were more episodic in nature. The nurses rarely visited more than once or twice a year. The infrequency of the visits served as a challenging condition for providing support to both the patients and caregivers. This made the nurses’ creativity and use of the opportunities arising in each situation even more crucial. The nurses had to make use of the information at hand to understand the particular challenges faced by each patient and caregiver. Despite this challenging condition, the conversations often included discussing experiences of existential issues, particularly depression and anxiety.

This study illuminates the complexity of supporting both patients and caregivers during healthcare encounters. By searching for transformative moments, nurses actively seek ways to relate to both patients and caregivers and support them in the individual yet shared relational process of reconciling with the deteriorating illness and adjusting to an altered life situation. Nurses help patients clarify their personal needs, as shown in Diana's case. However, arrangements for respite can also be legitimized, as in the case of Tom and his wife. In both cases, it is crucial to attend to patients and caregivers as individuals yet understand them in the scope of their relationship. Recent studies have described how the complex process of accommodating chronic illness affects marital relationships (Aasbø, Solbrække, Kristvik, & Werner, 2016; Hudson et al., 2016; Radcliffe, Lowton, & Morgan, 2013).

This article reveals the implications of the entangled, interdependent relationship between patients and caregivers for understanding their support needs. Several studies have pointed out that professional support often fails to recognize caregivers’ expertise and own support needs (Aasbø, Rugkåsa, Solbrække, & Werner, 2017; Røthing et al., 2015; Rugkåsa, 2015). The study findings indicate that the expertise, as well as support needs, patients and caregivers have vis‐à‐vis each other may create tensions between them. With few exceptions, illness management as a conflict between cared‐for and caregivers has received limited attention in the literature on informal care (Corbin & Strauss, 1984; Simpson et al., 2010). This study indicates that awareness and sensitivity to the tensions in illness management between patients and caregivers is crucial for providing tailored support for both.

### 4.3 Limitations of the study

This study's design uses an explorative, interpretative research approach. Our approach involves certain limitations to both the study's methodology and the findings. Although the interviewed nurses emphasized the importance of home visits, they tended to describe the interactions that took place with little specificity. Consequently, we developed the in‐depth understanding of how the nurses interacted face‐to‐face with both patients and caregivers from a limited number of observations of home visits. A further limitation of the study is that it lacks the subjective perspectives of patients and caregivers. We do not know how they experienced the nurses’ recognition and support. We have not examined home visits’ outcomes or effectiveness as therapeutic interventions for patients and caregivers; rather, we have studied and conceptualized nurses’ interactive approaches to this particular work context.

In line with our theoretical approach, we mainly based the portrayal of the home visits on how they were documented and reflected in the field notes. Mattingly's perspective offered a fruitful approach to exploring possibilities and challenges nurses face when helping patients and their caregivers find ways to understand and manage illness. The approach allowed us to see healthcare professionals and their interactions with patients with long‐term illnesses and their caregivers from a perspective that considers the importance of inter‐subjective recognition. Thus, supporting the complex relational processes of change and motivation involves enhancing mutual acceptance of and accommodations for illness between patients and their caregivers. However, a potential limitation of the study's analysis is that the categorizations and conceptualizations developed recognize only a few aspects of the support nurses provide to patients and caregivers. For instance, how nurses approach mental health issues in both patients and caregivers is not specifically addressed.

To present our main findings, we chose to share detailed sequences from only two home visits to provide an in‐depth understanding of how nurses interact with both patients and caregivers. We may have developed the insights from the interactions in these two visits at the expense of documenting the varieties and nuances of interaction in the remaining home visits. However, by presenting interactions where conflicts arose between the patient and caregiver, we hope to demonstrate how healthcare providers may use these opportunities to open up new understandings of illness management. The detailed sequences provide in‐depth descriptions of how nurses facilitate co‐constructions of new understandings and acceptance of a complex illness trajectory and an altered life situation. During other visits, we noticed instances where the nurses’ recognition did not open up the conversation or overlooked opportunities to promote transformative moments. Such observations confirmed our supposition that promoting transformative moments is challenging and demands disease‐specific competence, communicative and relational competences and an understanding of the implications of illness in the context of intimate relationships.

## 5 CONCLUSION AND RELEVANCE TO CLINICAL PRACTICE

This study provides new insights into the potential role of professional support in the context of developing sustainable methods for illness management in a marital relationship. Our analysis shows that ambulatory nurses make great efforts to create opportunities for change, such as greater acceptance of the conditions and motivations to make adjustments to accommodate a trajectory of deterioration. By acknowledging ambivalence, recognizing dilemmas and understanding conflicts between spouses, nurses are able to support both patients and caregivers. A more complex understanding of the informal caregiving relationship therefore is necessary to facilitate caregivers’ more explicit role in nursing practice. The concept of “transformative moments” can be used to extend our understanding of the role of professional support in chronic illness management. This study addresses the multifaceted professionalism required to give such support, which extends beyond disease‐specific knowledge to include relational competence and understanding. Conveying recognition of the challenges faced by both patients and caregivers may provide a valuable source of support for the caring relationship, as well as confirm patients’ and caregivers’ senses of self.

## CONFLICTS OF INTEREST

The authors of this study have no conflicts of interest to declare.
